# Uncommon Aetiology of a Posterior Circulation Stroke

**DOI:** 10.7759/cureus.93049

**Published:** 2025-09-23

**Authors:** Lawrence Truscott, Riaz Latif

**Affiliations:** 1 Hospital Medicine, Royal Devon University Healthcare NHS Foundation Trust, Barnstaple, GBR; 2 Stroke Medicine, Royal Devon University Healthcare NHS Foundation Trust, Barnstaple, GBR

**Keywords:** granulomatous inflammation, large vessel vasculitis, small lymphocytic lymphoma (sll), stroke, vertebral artery

## Abstract

In this case, a patient presented with sudden-onset diplopia and persistent dizziness. A CT of the head showed two areas of hypoattenuation, one within each cerebellar hemisphere. MRI of the head with diffusion-weighted imaging (DWI) was consistent with bilateral cerebellar infarcts, but there was no convincing evidence of vasculitis. A CT angiogram raised the possibility of vertebral artery dissection. However, an interventional neuroradiologist reviewed the imaging and thought a dissection and cerebral vasculitis were unlikely. Due to persistent inflammatory markers, a PET scan was performed. This confirmed large vessel vasculitis as the cause of her stroke. A weaning regimen of prednisolone and weekly methotrexate was started. Vasculitis is a rare cause of strokes and can lead to diagnostic uncertainty and a delay in treatment.

## Introduction

Strokes are defined by the National Institute for Health and Care Excellence (NICE) as a collection of signs and symptoms, thought to be secondary to vascular pathology, with resultant focal or global dysfunction of cerebral functions, lasting more than 24 hours [1].

In the UK, on average, there are around 100,000 strokes per year and ~1.3 million people living with a diagnosis of stroke. Strokes place a large burden on the National Health Service (NHS), with ~126,000 hospital admissions each year in England alone. Strokes are also a significant cause of morbidity and mortality in the UK, responsible for ~38,000 deaths every year. Interestingly, the age demographic of stroke onset has shifted over the nine years leading up to 2016. There has been an age reduction of 2.3 years in males and 1.5 years in females, with a third of all strokes occurring between the ages of 40 and 69 years old [1].

Strokes are subdivided into two categories: 85% are ischaemic and 15% are haemorrhagic [1]. The former is typically caused by a thrombus/embolic occlusion of one of the larger extra-/intracranial arteries, vertebral arteries or the smaller perforating arteries. Less common causes of strokes include antiphospholipid syndrome, Fabry’s disease, vasculitis, cerebral venous sinus thrombosis and dissection.

Vasculitis is an autoimmune condition which can affect small, medium and large blood vessels, both intracranially and extracranially. For this report, we will focus on large vessel vasculitis and its association with strokes. "Large blood vessel" refers to the aorta and its main branches. The incidence is around one to two people per 10,000 in the UK [2]. There are two main types: Takayasu’s arteritis and giant cell arteritis (GCA). These are characterised by chronic granulomatous inflammation of the aorta and its branches. GCA usually occurs in those over 50 years of age [3]. One review stated that involvement of the internal carotid arteries and vertebral arteries is uncommon [4]. This is further highlighted by a retrospective study, which identified nine patients with confirmed intracranial GCA over a 22-year period, with the vertebral artery being the second most affected [5]. Recognising this emphasises the rarity of this case.

## Case presentation

On September 11, 2024, a female patient in her 60s was referred to the ophthalmology department and the rheumatology clinic for suspected GCA. She reported having a headache for more than a week, which slowly started to localise to her right temple. The day prior to the clinic, she had transient visual changes when moving her head. She also reported general malaise and two occasions of drenching night sweats (on a background of previous small lymphocytic lymphoma (SLL)). She was found to have tenderness over her right temporal artery, possible thickening, and loss of pulse when palpated along the course of the artery. Consequently, she was treated for GCA with a weaning course of prednisolone. She had a temporal artery biopsy arranged, which was negative.

On February 13, 2025, she was due to be seen in the rheumatology clinic, but she cancelled as she felt unwell. Four weeks prior, she had a cough and fever, as well as loss of appetite.

On February 9, 2025, she experienced a sudden onset of dizziness and vomiting. The feeling of dizziness returned five days later and was present on standing or with any head movement. Furthermore, on the same day, she had a brief episode of diplopia. The impression was that this could be viral labyrinthitis, but a referral to ophthalmology and admission for further imaging was felt appropriate to rule out a posterior circulation stroke.

Her medical history included SLL, cervical intraepithelial neoplasia (grade two), GCA involving the right temporal artery, essential hypertension, osteoarthritis of the hand, and a positive diagnosis of COVID. Her sister had a history of vasculitis, and both her brother and mother were diagnosed with polycystic kidney disease.

The National Institute of Stroke Scale (NIHSS) score was one, as she only had mild central ataxia and a wide-based gait [6]; otherwise, the neurological examination was unremarkable.

Ophthalmology review showed no evidence of anterior ischaemic optic neuropathy.

**Figure 1:**
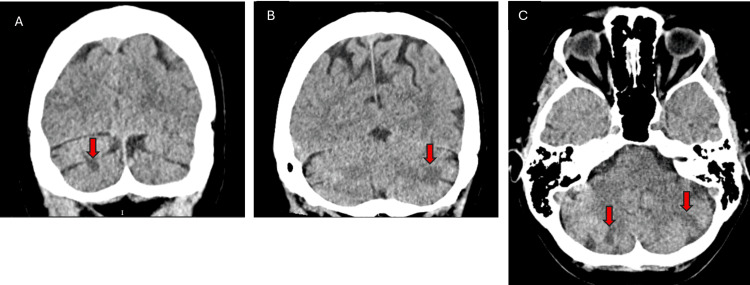
Coronal and axial sections of the head CT Images A and B are coronal sections of a CT of the head, and the red arrows demonstrate two separate areas of hypoattenuation within the right cerebellum and the left cerebellum, respectively. Image C is an axial section, and the red arrows illustrate two areas of hypoattenuation within each cerebellar lobe.

**Figure 2:**
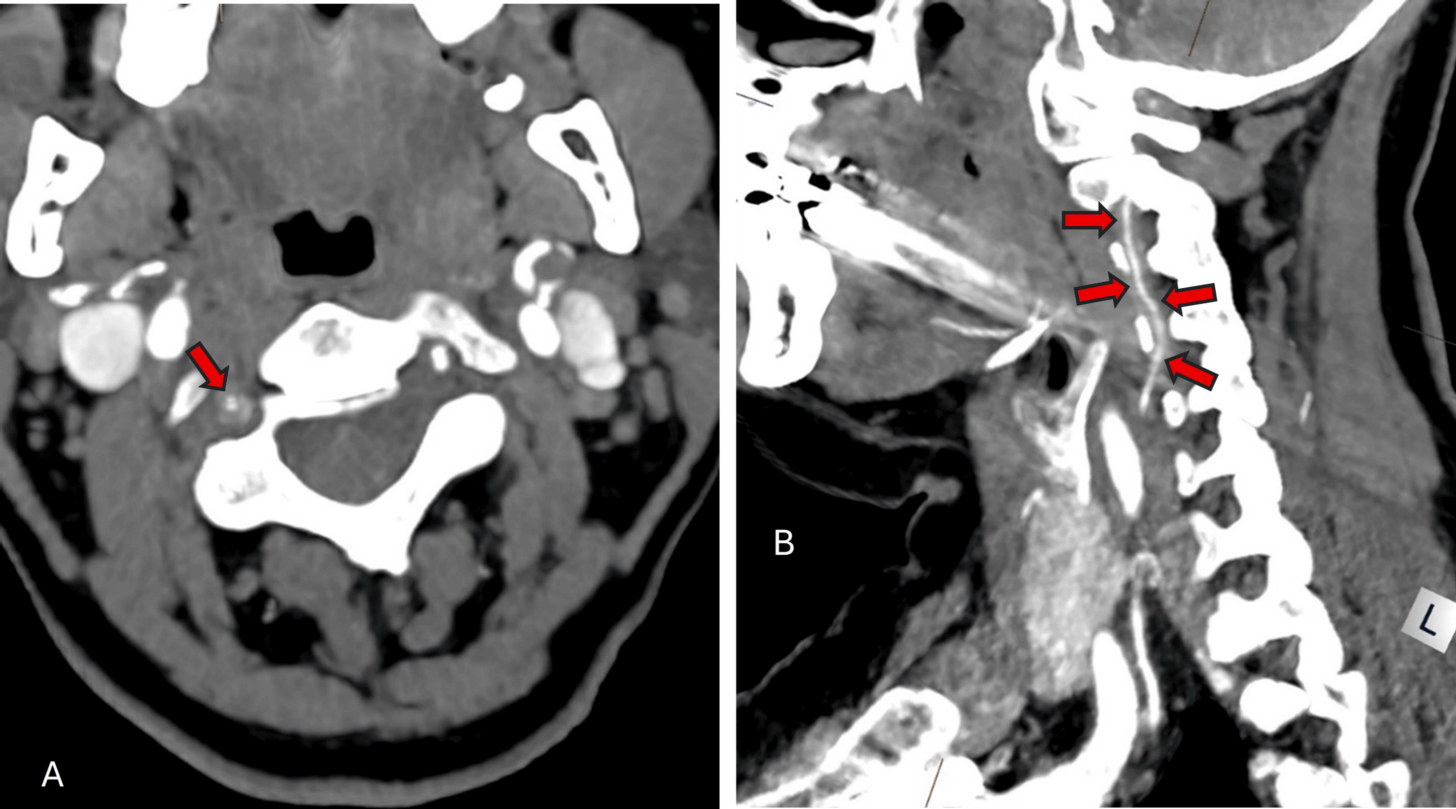
Axial and sagittal section of a CT angiogram Image A is an axial section of a CT angiogram and shows narrowing of the right vertebral artery. The red arrow indicates possible intramural thickening. Image B is a sagittal section of the left vertebral artery and its cervical course. Although difficult to appreciate, the red arrows are pointing towards the wall of the artery and again suggest that there may also be intramural thickening.

A CT of the head was performed, which showed hypoattenuation within both cerebellar hemispheres (Figure 1). Subsequently, an MRI of the head with diffusion-weighted imaging (DWI) showed restricted diffusion within both cerebellar hemispheres, consistent with bilateral cerebellar infarcts. A CT angiogram was requested to investigate for vertebral artery dissection. This report stated that at the craniocervical junction, there was reduced contrast opacification of both vertebral arteries, thereby raising the possibility of dissection (Figure 2). The images were reviewed by a consultant neuroradiologist who felt that vertebral artery dissection and vasculitis were unlikely. The patient was initially loaded with aspirin 300 mg and clopidogrel 300 mg, but following review by the stroke team, we decided to treat with just two weeks of aspirin 300 mg. Atorvastatin was increased to 80 mg. In addition, she was also treated with a course of doxycycline for a lower respiratory tract infection.

Given the ongoing clinical concern for vasculitis, an MRI of the head with contrast was requested. Furthermore, we requested this with T1 fat saturation to clarify whether there was a dissection. This scan did not show any evidence of dissection or vasculitis.

**Figure 3:**
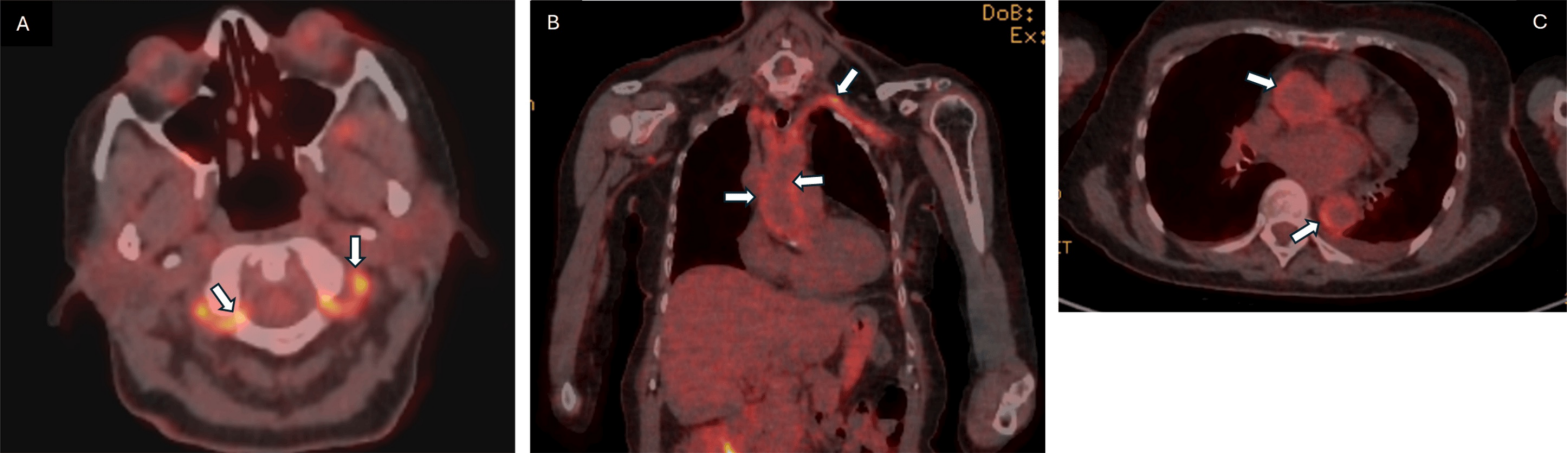
Axial and coronal sections of a fluorodeoxyglucose (FDG) PET scan Image A is an axial section of a FDG PET scan, and the white arrows illustrate increased activity within each vertebral artery. Image B is a coronal section. The white arrows indicate evidence of high uptake within the wall of the thoracic aorta, as well as the left subclavian, with extension into the axillary artery. Image C is an axial section which has been included to emphasise the increased activity within the wall of the ascending and descending aorta.

On February 16, 2025, the C-reactive protein (CRP) was still elevated at 142; therefore, a PET scan was requested. This showed widespread vasculitis affecting the aorta, subclavian, and vertebral arteries (Figure 3). This confirmed the aetiology of her stroke. Consequently, she was started on a weaning course of prednisolone and once-weekly methotrexate.

## Discussion

This case was not straightforward and presented several diagnostic challenges. Initially, the patient presented with constitutional symptoms prior to developing dizziness and vomiting; therefore, viral labyrinthitis seemed the most likely diagnosis. However, given the sudden onset of symptoms and recent treatment for suspected GCA, we investigated for a posterior circulation stroke.

The CT showed bilateral cerebellar infarcts, and this was confirmed with MRI. Vertebral artery dissection was considered; therefore, a CTA was requested. The report suggested that this could have been a possibility. However, despite sourcing a second specialist opinion, the diagnosis of both dissection and vasculitis remained uncertain. On closer inspection of the transverse and coronal sections of the CTA, there appears to be intramural thickening of the vertebral arteries (as displayed in Figures 3A, 3B).

We felt that she had a chest infection due to the chest X-ray findings and several pyrexic episodes; therefore, antibiotics were started. However, CRP remained persistently elevated, and this was likely due to the ongoing systemic inflammation from large vessel vasculitis. Given that CRP is non-specific, it should be interpreted in the context of the whole clinical picture, and clinicians should remember that there may be the presence of more than one pathology to explain its rise.

This illustrates how there were many confounding factors, which meant that a definitive diagnosis was delayed, and consequently, so was treatment.

One study focused on 287 patients over 27 years who had a confirmed diagnosis of GCA with temporal artery biopsy. Over this duration, eight patients suffered strokes, seven of which were within the vertebrobasilar region [7]. In a study of this duration, it illustrates the presence of this type of stroke (albeit rare) in this patient demographic and should be considered in those patients presenting with posterior circulation symptoms and elevated inflammatory markers with a recent diagnosis of GCA.

According to an update by the European Alliance of Associations for Rheumatology (EULAR) (2023), ultrasound (US) is now recommended as the initial imaging in suspected GCA and should include the axillary arteries [8]. US of both the temporal and vertebral arteries in GCA can reveal what is commonly known as the ‘halo sign’. This is caused by oedema of the intima, which is present prior to the development of stenosis. This has a high sensitivity and specificity for GCA [9]. The advantage of US as an imaging modality is that it is readily accessible, comparatively inexpensive and does not expose the patient to radiation. However, the hospital this patient attended is both rural and remote, and so one of our most significant limitations is the availability of sonographers who can perform and interpret such imaging. This patient had a temporal artery biopsy only, which was negative. 

In this case, Figure 3B demonstrates thickening of the vertebral artery along its course. Furthermore, the axial section in Figure 3A shows a narrowed vertebral artery and the appearance of intramural thickening. This explains why vertebral artery dissection was considered. However, CT imaging is not as useful when visualising the cranial arteries, and MRI/magnetic resonance angiography (MRA) or fluorodeoxyglucose (FDG)-PET may be a better option [10]. However, the MRI of the head with contrast that we performed did not show any evidence of vasculitis, but did refer to the vertebral arteries having a small luminal diameter. A systematic review and meta-analysis showed MRI to have a 73% sensitivity and 88% specificity when compared to a clinical diagnosis of GCA. Therefore, they concluded that MRI plays an important role in diagnosis [11]. A future consideration may be vessel wall MRI to identify areas of vascular inflammation. The drawback to this is that this type of imaging is highly specialised, costly and consequently limited to particular centres [12].

Large vessel vasculitis was confirmed with PET imaging, and as seen in Figures 4B, 4C, there is increased uptake of FDG within the thoracic aorta, as well as the subclavian and axillary arteries. Access to PET imaging is a limiting factor in diagnosis. The other drawbacks of this modality are that it is costly and exposes the patient to high levels of radiation. Furthermore, as part of treatment, patients usually take high-dose steroids. This can result in increased uptake of FDG by the liver and a decrease in mural uptake, meaning that the degree of activity may not be fully appreciated [13].

There does not seem to be a conclusive pathophysiological explanation of how large vessel vasculitis can lead to cerebrovascular events. Bonnan and Debeugny [9] proposed several different mechanisms to explain this. One being a temporal artery biopsy, which has shown granulomatous inflammation and intimal thickening; the presence of the latter results in a reduction in the diameter of the vascular lumen. If this affects cervical arteries in the same manner, then this may predispose patients to developing strokes in the future.

This patient had a diagnosis of SLL. There is a case report from 2015 about a 60-year-old gentleman who developed a rare case of large vessel vasculitis on a background of chronic lymphocytic leukaemia [14]. Therefore, there may well be an association between haematological malignancies and the development of vasculitis, albeit rare.

As a result of the immune-mediated response affecting the vessel walls of the major arteries, it makes sense that the treatment would be targeted towards immunosuppression and reducing inflammation. Therefore, following this patient’s confirmed diagnosis of large vessel vasculitis, a weaning regimen of prednisolone 40 mg once daily (OD) was initiated. The dose was to be reduced by 5mg each week to a maintenance of 20mg OD. Treatment for GCA had already been commenced five months previously, and now a stroke had manifested as a complication of large vessel vasculitis. Consequently, methotrexate 15mg once weekly was also started with a plan to increase to 20mg after two weeks, as well as folic acid 5mg every day, except for the day of methotrexate. The last review was a month later, and the patient reported that her symptoms had improved. However, the patient did experience some new symptoms, but these were attributed to steroid use. Consequently, the plan was to continue reducing prednisolone by 5 mg each week to a maintenance dose of 10 mg. Then continue 10 mg for four weeks, reducing by 1 mg a month thereafter. This is in line with current EULAR recommendations from 2018 [15].

To reduce this patient’s future stroke risk, secondary prevention was optimised. Consequently, atorvastatin was increased to 80 mg nightly. The HbA1c was within the normal range. Blood pressure was well maintained on amlodipine of 2.5 mg OD. The future management of this patient is complex and requires a multidisciplinary approach with input from the stroke services (including community neurorehabilitation), rheumatology, ophthalmology and their regular general practitioner.

## Conclusions

Large vessel vasculitis is a rare cause of strokes. Furthermore, a stroke as the initial clinical manifestation is even rarer. Therefore, if a patient is admitted with focal neurology on a background of recent presentations to rheumatology and has raised inflammatory markers, stroke secondary to vasculitis should be a consideration. The pathophysiology behind how large vessel vasculitis causes strokes is not well understood and warrants further understanding.

Each NHS trust will have its limitations with regard to access to imaging and available practitioners specialised in performing and interpreting these scans. A combination of CT and MRI will aid in determining the diagnosis of posterior circulation strokes caused by large vessel vasculitis. However, FDG-PET will clearly demonstrate those vessels with a high degree of activity. Future considerations include the use of vessel wall MRI, but this is costly, and as such, it is a limited resource.

Patients with a diagnosis of stroke and vasculitis require a multidisciplinary approach with input from the stroke services, including the neurorehabilitation team, rheumatology, ophthalmology (if vision is affected), and their general practitioner.

## References

[1] (2025). Stroke and TIA: causes. Clinical knowledge summaries. https://cks.nice.org.uk/topics/stroke-tia/background-information/causes/.

[2] Vasculitis UK (2025). Giant cell arteritis/temporal arteritis. https://www.vasculitis.org.uk/about-vasculitis/giant-cell-arteritis-temporal-arteritis.

[3] Gulati A, Bagga A (2010). Large vessel vasculitis. Pediatr Nephrol.

[4] Conticini E, Falsetti P, Bardelli M, Cantarini L, Frediani B (2021). Giant cell arteritis presenting as a stroke in the internal carotid artery territory: a case-based review. Reumatologia.

[5] Sanchez-Alvarez C, Hawkins AS, Koster MJ, Lehman VT, Crowson CS, Warrington KJ (2020). Clinical and radiographic features of giant cell arteritis with intracranial involvement. ACR Open Rheumatol.

[6] (2025). NIH Stroke Scale. https://www.ninds.nih.gov/health-information/stroke/assess-and-treat/nih-stroke-scale.

[7] Gonzalez-Gay MA, Vazquez-Rodriguez TR, Gomez-Acebo I (2009). Strokes at time of disease diagnosis in a series of 287 patients with biopsy-proven giant cell arteritis. Medicine (Baltimore).

[8] Dejaco C, Ramiro S, Bond M (2024). EULAR recommendations for the use of imaging in large vessel vasculitis in clinical practice: 2023 update. Ann Rheum Dis.

[9] Bonnan M, Debeugny S (2023). Giant-cell arteritis related strokes: scoping review of mechanisms and rethinking treatment strategy?. Front Neurol.

[10] Schmidt WA (2025). Biopsy vs imaging in the diagnosis of giant cell arteritis. Viewpoint 1: in favour of imaging. Rheumatology (Oxford).

[11] Duftner C, Dejaco C, Sepriano A, Falzon L, Schmidt WA, Ramiro S (2018). Imaging in diagnosis, outcome prediction and monitoring of large vessel vasculitis: a systematic literature review and meta-analysis informing the EULAR recommendations. RMD Open.

[12] Edjlali M, Qiao Y, Boulouis G, Menjot N, Saba L, Wasserman BA, Romero JM (2020). Vessel wall MR imaging for the detection of intracranial inflammatory vasculopathies. Cardiovasc Diagn Ther.

[13] Prieto-Peña D, Castañeda S, Martínez-Rodríguez I, Atienza-Mateo B, Blanco R, González-Gay MA (2021). Imaging tests in the early diagnosis of giant cell arteritis. J Clin Med.

[14] Kunnath TP, Rong R, Ambrus JL Jr (2015). Vasculitis associated with chronic lymphocytic leukemia. J Case Rep Stud.

[15] Hellmich B, Agueda A, Monti S (2020). 2018 Update of the EULAR recommendations for the management of large vessel vasculitis. Ann Rheum Dis.

